# Response to combination therapy of HCV 3a infected Pakistani patients and the role of NS5A protein

**DOI:** 10.1186/1743-422X-8-258

**Published:** 2011-05-25

**Authors:** Ijaz Ali, Sanaullah Khan, Sobia Attaullah, Shahid Niaz Khan, Jabbar Khan, Sami Siraj, Aqib Iqbal, Zahoor A Swati, Muhammad Idrees

**Affiliations:** 1 Institute of Biotechnology and Genetic Engineering, KP University of Agriculture, Peshawar, Pakistan; 2 Department of Zoology, Kohat University of Science and Technology, Kohat, Pakistan; 3 Department of Zoology, Islamia College Peshawar (A Public Sector University), University Campus Peshawar, Khyber Pakhtunkhwa, Pakistan; 4 Department of Biological Sciences, Gomal University, D.I.Khan, Pakistan; 5 Institute of Basic Medical Sciences, Khyber Medical University, Peshawar, Pakistan; 6 Centre of Excellence in Molecular Biology, University of the Punjab, Lahore, Pakistan

**Keywords:** HCV, genotype 3a, IFN, NS5A gene

## Abstract

**Background:**

Hepatitis C virus (HCV) genotype 3a is known to show comparatively better response to combination therapy than genotype 1 and 4. Mutations within NS5A gene of HCV have earlier been implicated with response to interferon (IFN) therapies in chronic HCV patients among various populations. As response to therapy are available in different populations because of the ethnic and viral factors and there was no study available on the phenomenon of resistivity to IFN.

**Results:**

Chronic HCV 3a infected Pakistani patients were kept on IFN-α and ribavirin therapy for six months. NS5A gene of HCV was amplified and sequenced in the case of all the patients prior to therapy and the sequences were analysed for mutations. Out of the total 27 patients, 20 (74.07%) were observed with sustained virological response (SVR), 4 (14.81%) patients were non responder (NR) while 3 (11.11%) patients exhibited in end of treatment response (ETR). Three (3/20) (15%) SVR patients and two (2/3) ETR patients had mutations (ranging from I-V amino acids) within the NS5A ISDR regions. While the rest of the SVR patients (85%) and the NR had no mutations at ISDR region when compared with HCV K3a ISDR.

**Conclusions:**

Mutations within the NS5A gene of HCV 3a genotype may not influence the outcome of combination therapy in Pakistani populations.

## Introduction

The global prevalence of Hepatitis C Virus (HCV) infection is 2.2%, corresponding to about 130 million HCV-positive persons worldwide [1]. Chronic hepatitis C viral infection can lead to cirrhosis and hepatocellular carcinoma [2]. The only drug that effectively reduces viral load is interferon-α (IFN-α) [3] and currently combination of IFN and ribavirin is the current treatment of choice [4,5]. HCV variants with mutations within the nonstructural protein 5A (NS5A) appeared to be more sensitive to therapy, suggesting that NS5A played a role in conferring IFN-resistance, thus this region was termed as IFN-sensitivity determining region (ISDR). Several studies showed that multiple mutations within ISDR are associated with IFN-α sensitivity in *HCV-1a/b *infected patients [6-9]. In patients infected with HCV genotype 3a isolates, no association between the number of mutations within the ISDR and treatment response was observed [10,11]. Recently, ISDR mutations with response to IFN-therapy in the case of HCV 1b have been correlated [12,13], but no such correlation was found in the case of HCV subtypes 3b and 1a [14].

Individuals infected with HCV genotype 1a/b show response rates of 38-52%, whereas 66-88% of those infected with genotypes 2 and 3 achieve sustained virological response (SVR). It is likely that the sensitivity or resistance to antiviral therapy is governed by both the virus and the host itself [15]. The clinical correlation between amino acid substitution within the NS5A ^2328-2376 ^including the ISDR according to *K-3a *[16] and response to antiviral treatment in HCV-3a infected patients; which is the predominant HCV genotype in Pakistan [17] is not as well known as for HCV-1a and HCV-1b infected patients. This study has attempted to elucidate the virological and biochemical aspects of HCV-3a infection, as well as to determine mutations in the *NS5A^2328-2376 ^*including the ISDR and correlate it with IFN-α and ribavirin standard therapy in chronically infected patients in Pakistan.

## Materials and methods

### Patients

Thirty patients (13 women, 17 men, average age 40 years (range 20-60 years) chronically infected with HCV subtype 3a and without any prior history of antiviral treatment were enrolled for this study. The estimated duration of infection varied from 6 months to 10 years (variable history) in different patients. The diagnosis of chronic HCV was based on elevated serum alanine aminotransferase (ALT) and aspartate aminotransferase (AST) levels for at least six months and consistent detection of serum HCV RNA. HCV antibodies (3^rd ^generation ELISA) were detected in each patient. All patients were negative for HBs Ag.

### Treatment

All the patients received 3 million IU of recombinant IFN-α three times weekly subcutaneously and ribavirin (10 mg/day/kg body weights) for a total of 24 weeks. Twenty-seven patients completed the therapy, while three of them including two male and a female patient opted to quit with it because of sever side effects. Informed consent was obtained from each patient.

### Patients Monitoring

In this study patients were monitored for HCV RNA presence and ALT level during and following treatment. Efficacy of treatment was assessed with normalization of ALT and absence of serum HCV RNA measured at week 12 and 24 and being undetectable at the end of the treatment at week 24 which constituted the end of treatment response and at the end of follow up at 12 or 24 months which constituted the sustained viral response for patients infected with HCV 3a. Doses of IFN were adjusted according to the platelet and white blood cells counts of the patient. Moreover, the doses of ribavirin varied according to the weight and the hemoglobin level of the individual patients.

### HCV RNA Extraction

HCV RNA was extracted from 100 μl serum sample by using RNA isolation kit (Gentra USA), according to the kit protocol, in Centre of Excellence in Molecular Biology, University of the Punjab, Lahore Pakistan. All the laboratory work was carried out in the same centre.

### HCV RNA detection, Quantitation and HCV Genotyping

Serum HCV RNA was detected by reverse transcription-PCR based on the highly conserved 5' untranslated genomic region for confirmation of HCV infection.

Quantification of HCV RNA was performed with real time PCR (Smart Cycler Cepheid USA). Genotyping of HCV was performed by type-specific PCR [18]. Qualitative and quantitative PCRs were repeated at various intervals of time during the treatment.

### Amplification of HCV NS5A cDNA by PCR

In HCV isolates of all HCV-3a-infected patients, the *NS5A *region was amplified after reverse transcription by nested PCR. The first round of PCR was performed using external sense primer OS5A-3a (5'-TACCGGACCCAGCACACCTTGCCCA-3'; nucleotide position 6555-6579 according to HCV-*K3a*) and antisense primer OA5A-3a (5'-GCTGAATTGTTCTTTTCCTCCGTGG-3'; nucleotide position 7336-7360). After an initial denaturation step at 95°C for 2 minutes, 30 cycles of 94°C for 45 seconds, 55°C for 45 seconds, and 72°C for 1 minutes was performed in a PE9700 thermal cycler (Perkin Elmer Cetus). In the second round of the nested PCR, 30 cycles using internal sense primer IS5A-3a (5'-TACTGAAGTGGATGGGGTGAGAATC-3'; nucleotide position 6723-6747 according to HCV-*K3a*) and the internal antisense primer IA5A-3a (5'-GGCGCATCCATGGACAGTTGGTGGT-3'; nucleotide position 7278-7302) was performed as described for the first round of the PCR. The resulting amplification product was analyzed on a 2% agarose gel stained with ethidium bromide.

### Sequencing

For direct sequencing of the *NS5A *gene of *HCV-3a *isolates including codons 2328-2376 according to HCV *K*3a, 20 μL each of the PCR products was purified from agarose gel by Pure Link™ Quick Gel Extraction Kit (http://www.invitrogen.com). Sequencing was performed according to the manufacturer's instructions (Big Dye Deoxy Terminators; Applied Biosystems, Weiterstadt, Germany). Sequencing of both positive and negative strands on automated sequencer (Applied Biosystems 310 DNA Sequencer) was carried out.

### Analysis of sequences

The HCV *NS5A^2328^*^-2376^protein sequences derived from the samples were aligned with HCV-K-*3a *prototype sequences and phylogenetic analysis was carried out with the help of GeneTyx for windows (Version 5.1).

### Nucleotide sequence accession numbers

NCBI accession numbers for *HCV K-3a *is **D28917 **and the nucleotide sequences of *NS5A^2328^*^-2376^of all groups of patients showing significant number of mutations were submitted to Genbank. Accession numbers of these sequences are DQ471944, DQ471945, DQ471946, DQ471947, DQ471948 and DQ471949.

## Results

### Confirmation of HCV 3a infection

To rule out the possibility of mixed infections with HBV or other HCV genotypes and to determine active HCV infection among the group members, initial qualitative PCR followed by genotype-specific PCR was carried out. It was confirmed that the patients selected were chronically infected with HCV 3a.

### Outcome of combination therapy

Of the 27 patients who completed antiviral treatment, 20 patients achieved sustained virological response (SVR) patients with undetectable HCV RNA 24 weeks after termination of therapy. In 3 patients a virological response with negative HCV RNA at the end of treatment but relapse thereafter was observed (ETR) patients. Four patients revealed no virological response (NR) to antiviral therapy at the end of treatment were observed.

### Analysis of mutations in the NS5A^2328-2376 ^region

**Figure 1:**
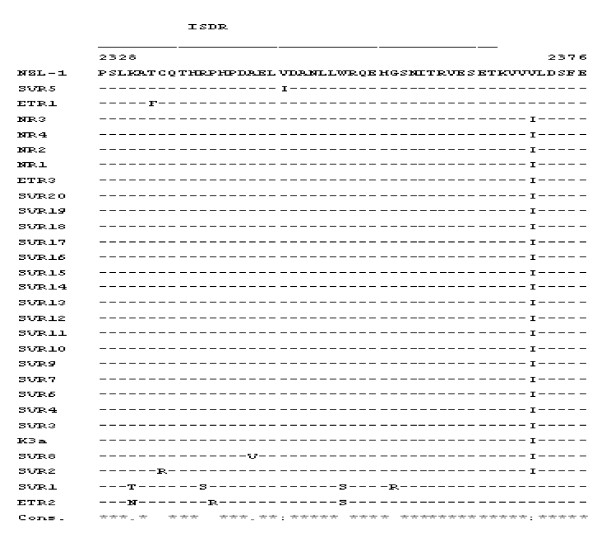
**Mutations within the *NS5A *of HCV 3a Pakistani isolates, the alignment of amino acid residues 2328 to 2376 compared with the reference prototype *HCV K-3a *sequences**.

After confirmation of HCV-3a infection in the case of all volunteers, *NS5A^2328-2376 ^*sequences (which include the ISDR) at various intervals of time during the therapy were analyzed. The mean number of mutations in SVR patients was 1.4 (range 1-5), 3 (1-5) in ETR patients and 1 in NR patients (Figure 1).

Out of 23 responders, 18 patients had a single mutation which was also shared by all the non responders. The remaining five samples had 2-5 mutations which were very much variable among all the five responders (SVR and ETR patients). Furthermore the two ETR patients with significant number of mutations had a relapse just within 12 months of the follow up period. Phylogenetic analysis of the isolates compared to HCV *K-3a *also did not show significant distances (Figure 1). We could not find any nucleotide mutations in the case of the non-responders and the ETR group patients, when the ISDR region was compared at the beginning, at 12 weeks or at 24 weeks of the combination therapy.

### Levels of viremia

In order to monitor primarily the response to IFN based therapy among 27 HCV-*3a *infected people; the viral load was measured at the beginning and during the course of the treatment. The initial viral load in SVR patients ranged from 5.03 to 5.68 log_10 _IU/ml, which dropped to the un-detectable level just after 12 weeks. In case of the ETR patients the viral load ranged from 5.35 to 5.56 log_10 _IU/ml. After 12 weeks, all the ETR group samples were positive for HCV RNA but with significant decrease in viral load. At the end of treatment, HCV RNA was not detected in these samples. The viral load of the non-responders showed a considerable decrease, but remained detectable throughout the time period of this study. Initially it ranged from 6.02 to 6.14 log_10 _IU/ml which fell down to an average 5.44 log_10 _IU/ml at the end of treatment.

### Enzymes Level

Assessment of the ALT enzyme level before treatment showed significantly higher values compared to a healthy control (5-40 U/liter). The mean value in responders was estimated to be 67.608 ± 12.2835 U/liter at the beginning of treatment, which eventually fell after treatment to 26.00 ± 4.9635 U/liter, which is within the normal range. Also, in non-responders, the mean value was found to be 72.75 ± 11.4419U/liter before treatment, which was reduced to 28.25 ± 3.304U/liter after treatment. Similarly, assessment of the AST level showed the mean value for the responders to be 74.8261 ± 14.4557U/liter, and that for non-responders was 80.00 ± 12.3828U/liter before treatment. Though, in both the cases, the enzyme levels were reduced to normal after treatment: responders showed 26.91 ± 8.857 U/liter, and for non-responders it was 29.25 ± 3.4034 U/liter.

## Discussion

Genetic heterogeniety has earlier been documented and the HCV genotype is the strongest predictive parameter for sustained virological response [19]. The NS5A gene of HCV has been reported to influence the outcome of IFN-based therapies in the case of people infected with various genotypes of HCV including HCV 3a [6,20,21]. Race and ethnicity have also been implicated to influence IFN responsiveness in chronic HCV patients [22]. HCV genotype 3a is the most abundant in Pakistan [23] and it has earlier been documented that HCV genotype 3a is more responsive to therapy as compared to genotype 1 or 4 [8,21]. Despite better response rate, the phenomenon of viral resistance is not uncommon in Pakistan. Initial viral load and biochemical profiles of chronic HCV patients also influence the outcome of therapy [24]. Earlier, in Pakistan, viral resistance in chronic HCV patients has never been investigated. The purpose of this study was to find out correlation if any of the IFN-responsiveness with mutations in the NS5A gene, initial viral load and the biochemical profiles of patients.

Our results indicated that out of the total 27 patients who had completed the six months combination therapy against HCV, 20 patients attained sustained virological response, 3 patients exhibited an end of treatment response while four patients were non-responders. The entire NS5A sequences of all HCV 3a isolates did not show any significant number of mutations to be correlated with treatment response in the case of different groups of patients. However, a number of mutations were found in the ISDR region of the NS5A protein and that is why the region was used for predicting response to therapy in Pakistani patients. When compared with HCV K3a prototype sequence, among the three ETR patients, ETR2 and ETR3 exhibited one mutation each in the ISDR region at position 2212 (K-N) and position 2244 (V-I) respectively. In the case of ETR1, In addition, both ETR2 and SVR1 also exhibited a (W-S) mutation at position 2233 while other mutations in the later including an (R-S), (G-R) and (K-T) mutations were observed at positions 2219, 2238 and 2212 of the ISDR region. Other mutations found in SVR patients include a (P-V) mutation in SVR8 at position 2224 and (V-I) mutation at position 2227. When compared with HCV K3a prototype sequence, SVR5 had another mutation (I-V) at position 2244 which was also found in the case of SVR1. Although a number of mutations were observed in the case of SVR and ETR patients, yet non were observed in the case of non-responders when compared with HCV K3a. The data indicates that the number of mutations found in SVR and ETR patients significantly correlates with the outcome of therapy in the case of SVR1, SVR5, SVR8 and the ETR patients but the fact that majority of the SVR patients share the same sequence as that of the non-responders rules out the possibility of these mutations playing a significant role with respect to viral resistance in chronic HCV patients of Pakistan. Other regional studies are in concordance with earlier findings [12,13]. In this study, neither the entire NS5A gene nor the ISDR region could be correlated with response to combination therapy in Pakistani HCV 3a infected patients.

The ALT levels and the initial viral load are also considered to predict response to therapy [24]. In this study, we observed that patients with lower viral titers before the start of treatment exhibited a significantly better response rate as compared to those with higher viral titers.

## Conclusion

Mutation in the NS5A gene of HCV genotype 3a may not affects the outcome of combination therapy in infected Pakistani patients. Mutations in the ISDR, along with mutations in other parts of the viral genome coupled with several other factors *e.g*. initial viremia, gender, may have a concerted effect on responsiveness to combination therapy.

## Authors' contributions

IA and MI designed and gave a critical view of manuscript writing. SK, SA and SNK helped in manuscript writing and data analysis. JK, AI, ZAS and SS gave critical view of manuscript writing and participated in data analysis. All the authors' read and approved the final manuscript.

## Competing interests

The authors declare that they have no competing interests.
